# Factors associated with the risk of malaria among children: analysis of 2021 Nigeria Malaria Indicator Survey

**DOI:** 10.1186/s12936-024-04939-6

**Published:** 2024-04-17

**Authors:** Isaac Isiko, Simon Nyegenye, Daniel Kiprotich Bett, Jackson Micheal Asingwire, Lenz Nwachinemere Okoro, Nana Awaya Emeribe, Catherine Chepkoskei Koech, Ovye Ahgu, Naya Gadzama Bulus, Kelly Taremwa, Aaron Mwesigwa

**Affiliations:** 1 Department of Community Medicine, Institute of Nutrition and Public Health, Nims University, Jaipur, Rajasthan India; 2 Department of Statistics and Applied Planning, School of Statistics and Planning, Makerere University, Kampala, Uganda; 3 Department of Radiation and Imaging Technology, Paramedical College, Nims University, Jaipur, Rajasthan India; 4 Faculty of Pharmacy, Marwadi University, Rajkot, India; 5 Department of Community Medicine, David Umahi Federal University Teaching Hospital, Uburu, Ebonyi State Nigeria; 6 Department of Community Medicine, Jos University Teaching Hospital, Jos, Nigeria; 7 Department of Sociology and Anthropology, Maseno University, Kisumu, Kenya; 8 Department of Public Health, Federal Medical Center, Keffi, Nigeria; 9 Department of Community Medicine, College of Medical Sciences, Abubakar Tafawa Balewa University, Bauchi, Nigeria; 10 School of Public Health, College of Health Sciences, Makerere University, Kampala, Uganda

**Keywords:** Children under 5 years, Family, Malaria, Nigeria, Malaria risk

## Abstract

**Background:**

Malaria remains a burden globally, with the African region accounting for 94% of the overall disease burden and deaths in 2019. It is the major cause of morbidity and mortality among children in Nigeria. Though different environmental factors have been assessed to influence the distribution and transmission of malaria vectors, there is a shortage of information on how they may influence malaria transmission among under-fives in Nigeria.

**Methods:**

This study was based on the secondary data analysis of the Nigeria Malaria Indicator Survey 2021. The study sample comprised 10,645 women (aged 15–49) who delivered a child in the 2 years preceding the survey. The study was restricted to under-fives. Logistic regression was used to identify factors associated with the risk of malaria.

**Results:**

There was a positive association between the risk of malaria and heard/seen malaria messages in the last 6 months (AOR 1.39, 95% CI 1.19–1.62), houses with walls built using rudimentary materials (AOR = 1.38, 95% CI 1.04–1.83), at least 6 children living in the house (AOR 1.22, 95% CI 1.00–1.49), children being 1 or 2 years old was associated with increased odds (AOR 1.89, 95% CI 1.50–2.34 and AOR 1.89, 95% CI 1.52–2.36), children from households with only treated nets (AOR 1.23, 95% CI 1.04–1.46) and those from the North West or South East regions (AOR 1.50, 95% CI 1.10–2.05 and AOR 1.48, 95% CI 1.01–2.16), respectively. All other predictors were not associated with the risk of malaria.

**Conclusion:**

The factors associated with the risk of malaria in this study included sleeping under treated mosquito nets, the age of the children, residing in the northwest and southeast regions, wall construction material, 6 children and above in the household and hearing/seen malaria messages in the last 6 months. Continuous health education and public health interventions, such as the provision of LLITNs, will reduce the risk of malaria and improve the health and well-being of children under 5 years of age.

**Supplementary Information:**

The online version contains supplementary material available at 10.1186/s12936-024-04939-6.

## Background

Malaria has been a serious global public health problem for several decades, especially in Africa and other endemic regions [1–3]. It is a fatal disease that is preventable and treatable and is transmitted to humans by female *Anopheles* mosquitoes [4, 5]. Malaria is an infectious disease caused by 5 protozoan species, namely; *Plasmodium falciparum*, *Plasmodium malariae*, *Plasmodium vivax*, *Plasmodium ovale*, and *Plasmodium knowlesi.* Among these species, *P. falciparum* is the most lethal pathogen and is found mostly in sub-Saharan Africa (SSA) [2, 3, 6, 7]. Generally, severe outcomes of malaria in children could result in seizures and coma, anaemia due to repeated infection and low birth weight during pregnancy, increasing the risk of death in the first month of life [8].

Malaria has continued to be the foremost cause of morbidity and mortality in children under 5 years old in sub-Saharan Africa [9, 10]. In 2022, globally, there were 249 million cases of malaria with 75% being children under 5 years of age [8]. Under-five children are more vulnerable to malaria infection than adults due to their lower immunity, thereby making them more susceptible to severe malaria due to repeated malaria infections [7]. A study in Nigeria showed that age, mosquito bed net usage, availability of health infrastructure, source of drinking water, distance to waste disposal points and window protection were significant determinants of malaria infection in children under 5 years of age [7, 11].

In 2020, 95% of all malaria cases and 96% of malaria deaths were reported in the African region with 80% of these deaths reported in children younger than 5 years. 6 sub-Saharan African countries, namely Nigeria (27%), the Democratic Republic of Congo (12%), Uganda (5%), Mozambique (4%), Angola (3.4%), and Burkina Faso (3.4%), bore 55% of the global malaria burden [12].

Nigeria has a high incidence of malaria mortality in children under 5 years, which is largely attributable to a health financing system that ignores uninsured individuals. This results in high out-of-pocket (OOP) medical expenditures that discourage healthcare-seeking behaviour, especially among these individuals [13]. The percentage of out-of-pocket expenditure medical expenditure in Nigeria in 2021 was 76% compared to 66% in 2005 [14]. The rate of care-seeking for suspected cases of malaria in Nigeria is among the lowest globally, as just under 20% of all children under 5 years with fever are taken for clinical consultation and parasitological testing in health facilities [13]. According to the Malaria Indicator Survey (MIS) 2021 in Nigeria, 22% of children tested positive for malaria parasites within 6–59 months according to microscopy results [15].

In the last two decades, many policies and interventions have been implemented to control malaria at the global and regional levels, which have resulted in a significant reduction in malaria-related morbidity and mortality [2]. The WHO Global Technical Strategy for Malaria 2016–2030 (GTS), the complementing Roll Back Malaria (RBM), and the Action and Investment to Defeat Malaria 2016–2030 (AIM) are notable malaria control initiatives that have been put into place in Nigeria and other malaria-endemic countries [16]. By 2030, they hope to eradicate malaria in 35 countries, decrease malaria mortality and case incidence by 90%, and stop the reintroduction of the disease into previously malaria-free areas [16].

However, despite these remarkable successes, there is still a high disease burden and mortality among children under 5 years of age [17]. This study aimed to identify the factors associated with the risk of malaria and to determine the predictors of malaria among children under 5 years in Nigeria.

## Methods

### Study setting and design

Nigeria is a country located in the western part of sub-Saharan Africa. The country is composed of 36 geopolitical regions, which include 36 states and one federal capital territory [18]. The country is divided into North Central, Northeast, Northwest, Southeast, South and Southwest regions, each with several states. Nigeria is neighboured by Benin to the west, Chad and Cameroon to the east, Niger to the north and the Gulf of Guinea of the Atlantic Ocean to the south.

The 2021 Nigeria Malaria Survey Indicator (2021NMIS) was constructed by the National Malaria Elimination Programme assisted by the National Bureau of Statistics (NBS) and National Population Commission (NPC) was implemented by the Global Fund to Fight AIDS, TB and Malaria (GFATM) [18]. The data were collected from 13/10/2021 to 4/12/2021.

The Nigeria Malaria Indicator Survey (MIS) data were collected in urban and rural areas from all 36 states, and the Federal Capital Territory (FCT) Nigeria is one of the tropical countries in West Africa and has the highest incidence of malaria in sub-Saharan Africa [18]. This study analysed data from the Nigeria Malaria Indicator Survey (NMIS) 2021, a nationally representative cross-sectional survey.

### Sampling and participants

The 2021 NMIS is the third malaria indicator survey conducted in Nigeria, with the first occurring in 2010 and the second occurring in 2015. The 2021 survey is unique in three ways. First, it was conducted in the first year of implementation of the current national malaria strategic plan.

A two-stage sampling strategy was adopted for the 2021 NMIS. In the first stage, 568 enumeration areas (EAs) were selected with a probability proportional to the EA size. The EA size is the number of households residing in the EA. The sample was selected in such a way that the sample was representative of each state. A total of 568 clusters were detected throughout the country, 195 in urban areas and 373 in rural areas. A complete listing of households in these clusters was (Additional file 1) conducted between 26 August and 18 September 2021, with the resulting lists of households serving as the sampling frame for the selection of households in the second stage. GPS dongles (global positioning system devices) were used to capture coordinates during household listing in the 2021 NMIS sample clusters. In the second stage of the selection process, 25 households were selected in each cluster via equal probability systematic sampling [18]. For the 2021 NMIS computer-assisted personal interviewing (CAPI) was used for data collection.

The study included women (15–49) years; each respondent provided informed consent to participate in the survey and understood the liberty of opting out any time she wished [18].

### Description of variables

The outcome variable was malaria status in a child, which was generated from the H71 (the respondent was told by the health work that the child had malaria) variable in the Women’s Health Questionnaire. Malaria was coded “has malaria” if the respondent was told that the child had malaria and “no malaria” if the respondent was told that the child had no malaria.

The Independent/predictor variables included socio-demographic characteristics namely house floor material, house wall material, roofing material of the house, mother’s age group, the main source of drinking water, mother’s number of living children, type of mosquito net in the household, sex of child, child age in years, type of place of residence, mother’s education”, combined wealth index.

## Results

### Characteristics of participants

A total of 10,645 under-five children were included in this analysis (Table 1). The majority were from households with improved sources of drinking water (71.93%), finished roofing (83.17%), finished walls (59.15%) and situated in rural areas (69.98%). More than half of the under-5 children did not have a mosquito net (56.92%) and were from the northern region (66.16%). This study included slightly more boys (51.24%) than girls (48.76%). Most mothers of these children had a maximum of 5 living children (81.32%), were between 25 and 34 years old (51.48%), and did not have access to the Internet the previous month (83.25%). Additionally, more than half of the young children under 5 years old were with mothers who had not received any malaria messages in the last 6 months (56.24%). Yet, most of these children were with mothers who had completed at least primary education (57.54%). The majority of the children under 5 years old were at least 2 years of age (61.33%). In most households, there were at most three under-five children (85.03%) and most of these children belonged to households with a mosquito net (63.57%).

**Table 1:** Malaria risk by socio-demographic factors among children under 5 years of age. Source: Nigeria Malaria Indicator Survey

Variable	Frequency (%), N = 10,645	Malaria n (%), total weighted% = 16.57	P-value
Heard/seen malaria messages in the last 6 months			< 0.001
No	5987 (56.24)	890 (14.87)	
Yes	4658 (43.76)	874 (18.75)	
Mother’s age group			0.296
15–24	2437 (22.89)	419 (17.18)	
25–34	5475 (51.48)	924 (16.89)	
35–49	2733 (25.67)	419 (15.33)	
Mother’s education			** < 0.001**
No education	4519 (42.45)	820 (18.14)	
Primary	1579 (14.83)	304 (19.23)	
Secondary ^+^	4547 (42.71)	621 (13.66)	
The main source of drinking water			** < 0.05**
Improved source	7657 (71.93)	1188 (15.52)	
Open source	2888 (27.13)	560 (19.40)	
Other	29 (0.27)	7 (24.84)	
Not a dejure resident	71 (0.67)	8 (11.48)	
Have mosquito bed net for sleeping in household			** < 0.001**
No	3878 (36.43)	496 (12.80)	
Yes	6767 (63.57)	1251 (18.48)	
Floor material			** < 0.001**
Natural floor	4497 (42.25)	867 (19.28)	
Rudimentary floor	85 (0.80)	7 (8.43)	
Finished floor	5978 (56.16)	860 (14.38)	
Other	85 (0.79)	13 (15.35)	
Wall material			** < 0.001**
Natural wall	905 (8.50)	144 (15.90)	
Rudimentary wall	3211 (30.16)	723 (22.53)	
Finished wall	6296 (59.15)	848 (13.48)	
Other	233 (2.19)	17 (7.10)	
Main roofing material			0.58
Natural roofing	1031 (9.69)	164 (15.91)	
Rudimentary roofing	597 (5.61)	109 (18.23)	
Finished roofing	8853 (83.17)	1472 (16.63)	
Other	164 (1.54)	15 (8.97)	
Frequency of using internet last month			** < 0.05**
Not at all	8862 (83.25)	1512 (17.06)	
Less than once a week	205 (1.93)	22 (10.96)	
At least once a week	439 (4.12)	69 (15.82)	
Almost every day	1139 (10.70)	154 (13.55)	
Number of living children			** < 0.01**
1–5	8656 (81.32)	1357 (15.68)	
6 and above	1989 (18.68)	400 (20.09)	
Sex of child			0.212
Male	5454 (51.24)	934 (17.12)	
Female	5191 (48.76)	830 (15.98)	
Child age in years			** < 0.001**
0	1919 (18.03)	225 (11.73)	
1	1969 (18.50)	381 (19.37)	
2	2025 (19.02)	400 (19.75)	
3	2095 (19.68)	328 (15.64)	
4	2409 (22.63)	396 (16.43)	
Type of mosquito net			** < 0.001**
No net	6059 (56.92)	860 (14.19)	
Only treated nets	4281 (40.22)	856 (20.0)	
Only untreated nets	129 (1.21)	25 (19.25)	
Region			** < 0.001**
North central	2024 (19.01)	261 (12.91)	
North east	1976 (18.56)	322 (16.32)	
North west	3043 (28.59)	650 (21.37)	
South east	1162 (10.92)	205 (17.69)	
South south	1396 (13.11)	175 (12.53)	
South west	1044 (9.81)	95 (9.05)	
Combined wealth index			** < 0.001**
Poor	4267 (40.08)	798 (18.70)	
Middle	2178 (20.46)	394 (18.11)	
Rich	4200 (39.46)	552 (13.15)	
Type of place of residence			** < 0.01**
Urban	3196 (30.02)	437 (13.66)	
Rural	7449 (69.98)	1415 (17.71)	

### Malaria risk according to sociodemographic factors among children under 5 years of age

From Table 1, the overall weighted percentage of contracting malaria was 16.57% among children aged 5 years of age. Remarkable differences were observed according to socio-demographic characteristics. The risk of contracting malaria was higher in rural areas (17.71%) than in urban areas (13.66%). This scenario was most risky in “Northwest” (21.57%), “South East” (17.69%), “North East” (16.32%), “North Central” (12.91%), “South-South” (12.53%) and least risky in “South West” (9.05%). The risk of malaria is higher among those whose mothers have no education (18.14%) or have completed only primary education (19.23%), compared to those whose mothers have attained at least secondary education (13.66%). The analysis also reveals the risk of malaria among children aged under five decreases with increase in women’s age group from those aged “15–24” (17.18%), “25–34” (16.89%), to “35–49” (15.33%). Households that reported others (24.84%) and open source (19.4%) as sources of drinking water were significantly associated with high malaria risk among children aged under five compared to those who reported Improved source (15.52%). Children aged 2 years and below are significantly associated with a high risk of malaria (50.85%) compared to those who are at least 3 years of age (32.07%). The study also shows highly significant malaria risk cases among those whose mothers did not use the Internet at all last month (17.06%) compared to those whose mothers reported “less than once a week” (10.96%), “at least once a week” (15.82%) and “almost every day” (13.55%), respectively. Households with at least 6 living children were significantly associated with high malaria risk (20.09%) compared to those that had “1–5” (15.68%) children in number. Additionally, chi-square tests showed that association between malaria risk and covariates heard/seen malaria messages in the last 6 months, mother’s education, main source of drinking water, having mosquito bed net for sleeping in household, floor material, wall material, frequency of using internet last month, number of living children, child age in years, type of mosquito net, region, combined wealth index and type of place of residence was statistically significant.

### Factors associated with malaria risk among children under 5 years of age

Results of the bivariate analysis are detailed in Table 2 with factors individually associated with malaria risk highlighted. In the final multiple logistic regression model, the factors found significantly associated with malaria risk were; heard/seen malaria messages in the last 6 months, wall material, number of living children, child age in years, type of mosquito net, and region. Children whose mothers received malaria messages in the last 6 months (AOR 1.39, 95% CI 1.19–1.62) were more likely to face malaria risk compared to those whose mothers did not receive such messages during that time. Those living in homes with rudimentary walls (AOR = 1.38, 95% CI 1.04–1.83) had higher odds of succumbing to malaria risk compared to those in homes with natural walls. Additionally, children of mothers with at least 6 living children (AOR 1.22, 95%CI 1.00–1.49) were more likely to face malaria risk than those with 1–5 living children. Being 1 or 2 years old was associated with increased odds (AOR 1.89, 95% CI 1.50–2.34 and AOR 1.89, 95% CI 1.52–2.36) of malaria risk compared to being newborn (0 years old). Moreover, children from households with only treated nets (AOR 1.23, 95% CI 1.04–1.46) and those from the North West or South East regions (AOR 1.50, 95% CI 1.10–2.05 and AOR 1.48, 95% CI 1.01–2.16) had higher odds of facing malaria risk compared to their counterparts from the North-central region.

**Table 2:** Factors Associated with malaria risk among children under 5 years of age

Variable	Crude odds ratio, P-value, COR (95% CI)	Adjusted odds ratio AOR, P-value (95% CI)
Heard/seen malaria messages in the last 6 months		
Yes	1.32** (1.14–1.54)	1.39** (1.19–**1.62)**
Ref = no	1	
Mother’s age group		
25–34	0.98 (0.84–1.15)	1.01 (0.85–1.19)
35–49	0.87 (0.71–1.07)	0.85 (0.67–1.07)
Ref = 15–24	1	1.00
Mother’s education		
Primary	1.07 (0.84–1.15)	1.16 (0.88–1.53)
Secondary +	0.71** (0.61–**0.84)**	0.95 (0.73–1.23)
Ref = no education	1	1.00
The main source of drinking		
Open source	1.31* (1.05–1.64)	1.18 (0.92–1.53)
Other	1.79 (0.33–9.77)	2.80 (0.65–12.17)
Not a *de jure* resident	1.79 (0.33–9.77)	1.82 (0.26–12.66)
Ref = Improved source	1	1.00
Have mosquito bed net for sleeping		
Yes	1.54** (1.29–1.85)	1.14 (0.93–1.40)
Ref = no	1	1.00
Floor material		
Rudimentary floor	0.39 (0.13–1.13)	0.62 (0.19–2.00)
Finished floor	0.70** (0.59–0.83)	1.03 (0.80–1.32)
Other	0.76 (0.31–1.94)	1.98 (0.70–5.59)
Ref = natural floor	1	1.00
Wall material		
Rudimentary wall	1.54** (1.16–2.04)	1.38* (1.04–**1.83)**
Finished wall	0.82 (0.63–1.08)	0.76(0.56–1.05)
Other	0.40* (0.18–0.88)	0.37* (0.15–**0.92)**
Ref = natural wall	1	1.00
Main roofing material		
Rudimentary roofing	1.18 (0.67–2.08)	0.95 (0.55–1.62)
Finished roofing	1.05 (0.72–1.55)	1.14 (0.75–1.75)
Other	0.52 (0.22–1.25)	0.66 (0.23–1.94)
Ref = natural roofing	1	1.00
Frequency of using the internet last month		
Less than once a week	0.59 (0.3–1.04)	0.84 (0.46–1.54)
At least once a week	0.91 (0.68–1.22)	1.29 (0.95–1.76)
Almost every day	0.76* (0.58–**0.99)**	1.22 (0.90–1.65)
Ref = not at all	1	1.00
Number of living children		
6 and above	1.352** (1.13–**1.62)**	1.22* (1.00–**1.49)**
Ref = 1–5	1	1.00
Sex of child		
Female	0.92 (0.81–1.05)	0.94 (0.82–1.07)
Ref = male	1	1.00
Child age in years		
1	1.81** (1.4–**2.28)**	1.89**(1.50–**2.34)**
2	1.85** (1.49–2.29)	1.89** (1.52–**2.36)**
3	1.39** (1.13–1.73)	1.45** (1.17–**1.79)**
4	1.48** (1.19–1.84)	1.49** (1.20–**1.87)**
Ref = 0	1	1.00
Type of mosquito net		
Only treated nets	1.51** (1.31–1.75)	1.23* (1.04–**1.46)**
Only untreated nets	1.44 (0.82–2.54)	1.30 (0.74–2.29)
Ref = no net	1	1.00
Region		
North east	1.32 (0.88–1.96)	1.13 (0.75–1.72)
North west	1.83** (1.35–2.49)	1.50* (1.10–**2.05)**
South east	1.45* (1.01–2.07)	1.48* (1.01–**2.16)**
South south	0.97 (0.67–1.39)	1.14 (0.79–1.64)
South west	0.67* (0.46–0.99)	0.77 (0.53–1.12)
Ref = north central	1	1.00
Combined wealth index		
Middle	0.96 (0.78–1.18)	1.23 (0.93–1.63)
Rich	0.66** (0.55–0.79)	1.08 (0.74–1.58)
Ref = poor	1	1.00
Type of place of residence		
Rural	1.36** (1.13–1.63)	1.01 (0.83–1.24)
Ref = urban	1	1.00

^*^p < 0.05, **p < 0.01

## Discussion

This study identified factors associated with the risk of malaria among children under 5years of age. Children whose mothers had been exposed to malaria messaging within 6 months before the study showed a heightened risk of contracting malaria compared to those whose mothers hadn’t received such information. This paradox highlights the complex interplay between information dissemination, health literacy, and disease prevention. While access to information is crucial for empowering parents to protect their children from malaria, this study suggests that the timing or content of the message may have unintended consequences.

Additionally, the study revealed a correlation between housing quality and malaria risk among children. Those residing in homes with rudimentary wall materials faced elevated risks compared to those in dwellings with natural materials, emphasizing the critical role of housing standards in disease transmission, particularly for malaria. This finding echoes similar observations from studies conducted in Uganda, emphasizing the broader implications for malaria control strategies [14]. Improving housing conditions not only addresses individual health but also contributes to broader public health objectives by reducing malaria transmission rates within communities.

Furthermore, the study identified an unexpected association between malaria risk and the use of treated mosquito nets. Contrary to expectations based on previous studies demonstrating significant protection conferred by insecticide-treated nets (ITNs), children who exclusively used treated nets showed higher chances of malaria infection [19]. This suggests a potential over-reliance on ITNs as the sole preventive measure, neglecting other malaria prevention strategies. It underscores the importance of comprehensive malaria prevention education, emphasizing the complementary nature of various preventive measures. Targeted efforts to educate the public on adopting multi-pronged approaches to malaria prevention are crucial for maximizing the effectiveness of interventions and reducing the malaria burden in vulnerable populations.

This study further unveils additional factors intertwined with the risk of malaria, providing crucial insights with significant public health implications. Among these factors are the number of living children in a household and the age of the child. Households with 6 or more living children were found to have an increased risk of malaria compared to those with one to 5 children. This finding underscores the intricate relationship between family size, household dynamics, and malaria transmission [20]. Larger families may experience heightened population density and overcrowding, potentially leading to challenges in implementing effective malaria control measures. The strain on basic amenities in such households could contribute to environmental neglect, creating more breeding grounds for mosquitoes and amplifying the risk of malaria transmission. This highlights the importance of tailored interventions addressing the specific needs of larger families, including enhanced access to and compliance with malaria prevention measures. This increased risk associated with larger families aligns with findings from studies examining the impact of urbanization and population density on childhood *Plasmodium falciparum* parasite prevalence rates in Africa [20]. The correlation between household size and malaria risk underscores the broader implications of socio-demographic factors in shaping malaria transmission dynamics within communities. Addressing these underlying socio-economic determinants is essential for developing holistic malaria control strategies that effectively target vulnerable populations and reduce transmission rates.

In a contrasting observation, children aged 1 and 2 years were found to have an increased risk of malaria compared to those younger than 1 year old. This phenomenon may be attributed to the possession of immunity acquired from mothers during infancy, as well as the increased care and attention typically given to newborns. However, as children grow older, this acquired immunity wanes, leaving them more susceptible to malaria infection. This highlights the importance of age-specific interventions in malaria control efforts, ensuring that preventive measures are tailored to the unique vulnerabilities and immunity profiles of different age groups.

This study sheds light on a complex of factors influencing malaria risk in children under 5, unveiling both anticipated and surprising associations [14]. While access to information and preventative measures like treated mosquito nets are crucial, their timing, content, and implementation require meticulous approaches to maximize effectiveness. The study underscores the interplay between social, environmental, and demographic factors, emphasizing the need for holistic and multifaceted interventions that address not only individual risk but also underlying socio-economic determinants within communities. Tailored interventions for larger families, age-specific strategies, and comprehensive education fostering multi-pronged approaches are key to reducing the malaria burden and empowering vulnerable populations [19]. This study’s insights, echoing similar observations from broader contexts, pave the way for evolving malaria control strategies that effectively target risk factors and ultimately break the cycle of transmission, safeguarding the health and well-being of children.

### Limitations of the study

A two-stage sampling strategy was used during data collection but in our analysis, we didn’t take into consideration the hierarchical levels which could affect the interpretation of our results. The study was purely based on data from children under 5 years hence the findings cannot be used to conclude other groups of children such as children older than 5 years within the Nigerian communities.

### Supplementary Information

**Additional file 1.** Covariates.

## Data Availability

The dataset used is openly available upon permission from the MEASURE DHS website. (URL: https://www.dhsprogram.com/data/available-datasets.cfm).

## Abbreviations

GPS: Global positioning system
COR: Crude odds ratio
AOR: Adjusted odds ratios
LLIN: Long-lasting insecticidal nets
WHO: World Health Organization
NMIS: Nigeria Malaria Indicator Survey
CI: Confidence interval
NMEP: National malaria elimination programme
DHS: Demographic and Health Survey
SDSS: Spatial decision support systems
FMOH: Federal Ministry of Health
NBS: National Bureau of Statistics
PMI: Presidential malaria initiative
GFATM: Global fund to fight AIDS, TB and malaria
SSA: Sub-Saharan Africa
FCT: Federal capital territory
CAPI: Computer-assisted personal interviewing
NPC: National population commission
IBR: Institutional Review Board
USAID: United States agency for international development
NMSP: National malaria strategic plan
RBM: Roll back malaria
RDT: Rapid diagnostic test
malERA: Malaria eradication research agenda
AIM: Action and investment to defeat malaria 2016–2030
ITNs: Insecticide treated nets
