# Supplementary material for: Factors associated with the risk of malaria among children: analysis of 2021 Nigeria Malaria Indicator Survey
Source: Malar J. 2024 Apr 17;23:109. doi: 10.1186/s12936-024-04939-6 (PMC11025242; doi:10.1186/s12936-024-04939-6)
Supplement: Supplementary file 1 — Additional file 1. Covariates. [file 12936_2024_4939_MOESM1_ESM.docx]

**Covariates**

The covariates selected in the study included socio-demographic characteristics namely floor material (“natural floor”, “rudimentary floor”, “finished floor” and “other” ), wall material (“natural wall”, “rudimentary wall”, “finished wall” and “other” ), roofing material (“natural roofing”, “rudimentary roofing”, “finished roofing” and “other”), mother’s age group (“15 – 24”, “25 - 34” and “35 – 49”), the main source of drinking water (“improved source”, “open source”, “other” and “not a dejure resident”), mother’s number of living children (“0”, “1 – 5”, “6 and above”), type of mosquito net in the household (“no net”, “only treated nets ”and “only untreated nets”), sex of child (“male” and “female”), child age in years (“0”, “1”, “2”, “3” and “4”) and type of place of residence (“urban” and “rural”). In this study, the mother’s education for secondary and higher was merged as “secondary+”; both no education and primary were retained as “no education” and “primary”, combined wealth index (“poor”, “middle” and “rich”) respectively
